# Context shapes evaluation of emotional valence, not emotional categorization: A fresh look at the Kuleshov effect

**DOI:** 10.1177/20416695251410119

**Published:** 2026-03-09

**Authors:** Bence Szaszkó, Mark Andrej Loebus

**Affiliations:** 1 Public Mental Health Research Unit, Department of Social and Preventive Medicine, 27271 Medical University of Vienna, Austria; 2 Department of Cognition, Emotion, and Methods in Psychology, Faculty of Psychology, University of Vienna, Austria

**Keywords:** context, emotion attribution, facial expression, Kuleshov effect

## Abstract

In filmmaking, a neutral face paired with an emotional context can convey a congruent emotion—a phenomenon known as the Kuleshov effect. However, past research has yielded mixed findings on the existence of the Kuleshov effect, possibly due to methodological variability; we therefore implemented a boundary-test paradigm that combined normed static context images with dynamic facial stimuli separated by an explicit context-rating step to examine how visual context shapes the evaluation and categorization of faces under such conditions. We hypothesized that positive context would elevate facial valence ratings (and vice versa), while evoking different-than-neutral emotions in a neutral face when categorized. Thirty-two participants first rated the valence of a context image on a 7-point Likert scale. They then viewed a video of a neutral face, evaluated its valence on the same scale, and explicitly categorized the expression by selecting one of five predefined emotion labels (neutral, angry, happy, sad, disgusted). Analyses using linear mixed-effects models confirmed that positive contexts led to higher facial valence ratings, while negative contexts led to lower ones; by contrast, multinomial regression revealed no effect of context on emotional categorization. Our findings suggest that while visual context can bias evaluative judgments of facial expressions, it does not necessarily alter their emotional categorization under conditions where cinematic continuity is absent, highlighting the boundaries of the Kuleshov effect in line with previous studies.

## How to Cite this Article

Szaszkó, B., & Loebus, M.A. (2026). Context shapes evaluation of emotional valence, not emotional categorization: A fresh look at the Kuleshov effect. *i–Perception*, *17*(2), 1–13. https://doi.org/10.1177/20416695251410119

## Introduction

Films aim to tell stories, convey messages, and entertain audiences; they achieve this most effectively by presenting emotionally charged scenes that move the viewer (Prince, 1997). One powerful tool in this process is the use of a close-up of a face. However, a close-up alone does not correlate with emotional responses; it is the combination of multiple shots that creates this impact (Benini et al., 2022). Editing is crucial for creating context, conveying emotions, and shaping the film's visual grammar. In this context, assigning meaning to a neutral or ambiguous facial expression by providing visual context through editing can be particularly useful.

Contextual information—verbal, visual, and auditory—serves as an interpretive aid, particularly for neutral and ambiguous facial expressions (Wieser & Brosch, 2012). Objects co-occurring with a face can, for instance, flip its perceived valence from pleasant to unpleasant, while the same face can be relabeled corresponding to a completely different emotion when contextual cues change—an effect that emerges already at early perceptual levels (Aviezer et al., 2008). Comparable results are achieved by affective sounds that accompany an otherwise neutral face (de Gelder & Vroomen, 2000), showing how emotion perception can flexibly integrate a wide range of contextual information.

A phenomenon in filmmaking that supposedly capitalizes on the manipulation of visual contextual information is the Kuleshov effect, which demonstrates that the interpretation of a neutral facial expression can be influenced by the emotional context of preceding or accompanying visual scenes. The Kuleshov effect was first explored in the 1910s by film theorist Lev Kuleshov and his protégé Vsevolod Pudovkin in an experiment (Pudovkin, 1970). The original Kuleshov experiment featured a neutral face of an actor paired with scenes of a deceased woman, a bowl of soup, and a girl playing. Reports suggest that audiences interpreted the identical facial expression depending on the preceding scene—associating it with grief, hunger, or joy (Isenhour, 1975). Notably, the original film and detailed accounts of the experiment's procedure were lost, leading to its perception as a cinematic urban legend rather than a verified psychological phenomenon (Holland, 1989), and raising concerns about the validity, reliability, and even the actual execution of the experiment. Nevertheless, the Kuleshov effect has since become a foundational example in cognitive film theory of how viewers actively infer internal states based on cinematic cues: Rather than passively receiving emotional meaning, spectators integrate context, framing, and prior knowledge to mentally simulate what the character is feeling or thinking, aligning with Bordwell's (1985) account of the film viewer as an active information processor in terms of narrative comprehension. At the same time, other work has highlighted the specifically emotional and empathic dimensions of this process: Carroll (1996) analyzed how point-of-view editing communicates and modulates viewers’ emotions in a way that they align with the character's perspective, shaping their emotional responses; in line with this argumentation, Smith (2022) investigated how film engages viewers by inviting empathetic responses to characters, and Plantinga (2009) elaborated how film form influences the affective experience of viewers. Consistent with this account, it has been shown that visual context influences the perception of valence, arousal, and emotions in faces, potentially by creating expectations of a particular facial expression and thereby suggesting an interpretation of the actual facial expression, or by distortion of facial expressions through empathy triggered by the visual context (Barratt et al., 2016). If the emotion of the context matches the emotion of the facial expression, the emotion is perceived more intensely (Calbi et al., 2019). Together, these accounts underline that the Kuleshov effect is not only a matter of cognitive inference but also closely tied to how cinema orchestrates emotion and empathy.

Early replication attempts produced conflicting evidence for the Kuleshov effect. Prince and Hensley (1992) found no influence of visual context on the interpretation of a neutral face, while Wallbott (1988) identified a significant effect of visual context on emotion perception using film and television clips. Barratt et al. (2016) found that participants rated the same neutral face differently depending on the emotional valence of the preceding image, suggesting that presenting a consistent spatial scene and having the face appear to look at something within that scene may help viewers more easily connect the emotional meaning of the context with the face. In an experiment by Calbi et al. (2017), the interpretation of faces was significantly altered when crosscut with a short film scene that could be happy, neutral, or fearful. Follow-up studies by Calbi et al. (2019) further demonstrated that these effects are also partly reflected in neural activity, implicating brain regions involved in emotional and facial processing. Multiple further studies using functional magnetic resonance imaging were also able to provide evidence that the Kuleshov effect existed not only in subjective emotional perception but also at the neural level (Cao et al., 2024; Mobbs et al., 2006). Of note, applying still photographs in a standardized setting also resulted in a contextual effect on emotion perception and an influence of negative context on facial valence, even when the context and face stimuli were temporally separated (Mullennix et al., 2019). The Kuleshov effect was also replicated in a multisensory experiment by Baranowski & Hecht (2017), in which background music significantly biased the emotion attributed toward a face, implying that contextual valence is sufficient to influence emotional perception.

While some of the prior studies (Barratt et al., 2016; Calbi et al., 2017, 2019) were conducted within a unified research agenda, with shared stimuli and methodology, procedural inconsistencies with other studies may still contribute to the mixed findings reported across experiments. Capitalizing upon the merits of these studies, we opted for a somewhat different approach aimed at examining the effect of visual context on the interpretation of facial expressions and emotions using short videos for all neutral facial expressions: Our design was largely inspired by the study of Mullennix et al. (2019) who opted for a dyadic (context, then face) rather than a triadic (context, face, second face appearing as a reaction) trial structure. Because that extra reaction can itself convey emotion, we share the opinion of Mullennix et al. (2019) that a dyad provides the stricter test of whether mere framing, without an explicit reaction, can bias perception. Unlike in classic demonstrations of the Kuleshov effect, we also included a context rating to ensure deliberate processing of the corresponding scene, potentially maximizing the emotional impact of context while still leaving facial evaluation entirely implicit (see again Mullennix et al., 2019).

Notably, the structure of our paradigm significantly differs from cinematic realism traditionally used in Kuleshov-type sequences; as Barratt et al. (2016) highlight, viewers often interpret Kuleshov-type sequences as continuity edits involving the evoking of the impression of an actor reacting to something in shared space. On the contrary, in our paradigm, faces look directly at the viewer, no return shot is included, and context and face are separated temporally. By adopting a dyadic structure and breaking with continuity, our approach allows us to isolate the influence of visual context on facial evaluation without additional confounding effects, assessing perceptual bias in an isolated manner. This approach, however, may render our paradigm a boundary test rather than a full replication of the Kuleshov effect (with this limitation explicitly addressed in the Discussion).

We first assumed that the more positive (negative) the context, the more positive (negative) the valence evaluation of neutral facial expressions (H1). In line with previous studies, we also hypothesized that visual context influenced the explicit categorization of emotions in neutral facial expressions (H2). This hypothesis is considered confirmed if an emotion is attributed to the neutral facial expression based on the context during the process of explicit categorization (see below). Together, H1 and H2 test the same overarching construct at two complementary levels—with valence as a continuous dimension and emotion category as a nominal choice.

## Method

A power simulation using custom Python code indicated that 29 participants were needed to achieve 90% power at an alpha level of .05 and an effect size of *d* = 0.5, with past studies producing even larger than medium effect sizes. 32 healthy psychology students (24 female, *M*_Age_ = 22.22 years, *SD*_Age_ = 3.78 years) from the University of Vienna participated in the study in exchange for partial course credit. Participants had to be at least 18 years old and were required to have no history of psychological or psychiatric disorders. Current daily mood was included as a level-1 control variable, while age, gender, and sexual orientation were included as level 2 control variables.

### Materials

We selected three sets of 54 prerated photos—classified as positive, neutral, and negative based on valence ratings—from the Open Affective Standardized Image Set (OASIS; Kurdi et al., 2017) database as context stimuli. The selection of context photos was carried out in several steps. First, we sorted all 900 photos by their mean valence scores and standard deviations. Next, we calculated the distance of each photo's valence score from the median. For the neutral context group, we selected the 54 photos closest to the median. Then, we selected 54 photos randomly from the upper third of the valence distribution for the positive context group. Each positive context photo was paired with a counterpart from the lower third of the valence distribution that had a similar distance from the median. These 54 photos constituted the negative context group. Positive context photos depicted animals, people, and nature, such as dogs, musicians, and beaches. The negative context consisted of images of people, acts of violence, and photos evoking disgust reactions, including crying babies, soldiers, and surgeries. The neutral context group featured photos of objects, animals, and people, such as rocks, pigs, and students. We took special care to avoid images depicting nudity, explicit violence, or other potentially disturbing content to ensure that participants were not unintentionally exposed to emotionally distressing or inappropriate imagery.

We produced neutral facial expression videos specifically for this study. These videos were prescreened by seven independent raters, who rated their valence and categorized the displayed emotion. The emotional categories included “happy,” “sad,” “neutral,” “fearful,” and “disgusted” (adopted from Mullennix et al., 2019). Only facial expressions that were predominantly rated as neutral during prescreening were used, with the nine female and nine male faces that were most frequently rated as neutral selected for further use. The videos were filmed using the same camera under similar lighting conditions. The faces were filmed facing the camera directly to avoid the appearance that the actors were looking at the context image. Gender balance was ensured in the actor selection. From 25 neutral facial expressions in total, the nine most neutral male and the nine most neutral female faces were selected for the experiment.

### Apparatus, Stimuli, and Procedure

In previous studies, the Kuleshov experiment typically followed a specific structure: a visual context stimulus was presented, followed by a facial expression, which participants were then asked to evaluate. However, Mullennix et al. (2019) pointed out several issues in these earlier experimental designs. For instance, although several researchers such as Barratt et al. (2016) and Calbi et al. (2017, 2019) took care to validate their stimuli, in earlier studies (e.g., Prince & Hensley, 1992; Wallbott, 1988), the context stimuli were repeatedly not standardized and were selected and presented arbitrarily by the researchers without being prescreened. Additionally, while the study by Barratt et al. (2016), as well as the studies by Calbi et al. (2017, 2019) used a standardized 3-s context presentation consistent with cinematic norms to ensure sufficient time to process the context image that was immediately followed by the facial expression, Wallbott (1988) used considerably longer clips with high variation (around 16 s for context and 5 s for faces), while Prince & Hensley (1992) presented context stimuli for a duration of 7 s. However, such processing may have been necessary to ensure participants’ attention to the context image. Therefore, we adopted participant-controlled timing and an explicit context rating as a precautionary measure, as we considered it preferable to err on the side of caution and directly ensure that participants engaged with and interpreted the context stimuli as intended. To address these shortcomings, our study was based on the experimental design proposed by Mullennix et al. (2019) for investigating the Kuleshov effect, which introduced solutions to these problems. Similar to their study, the context images in this experiment were sourced from a standardized database with validated valence and arousal ratings. Participants could decide how long to view each context image, provided a minimum viewing time was met, and then evaluated the context image. This ensured that participants’ attention was directed toward the context stimulus.

The experiment was created and run using PsychoPy software (Version 2024.1.1, Peirce et al., 2019) on a G2590PX AOC Gaming LCD monitor with a screen size of 24.5 inches and a maximum refresh rate of 144 Hz in a dimly lit room. Before the experiment, participants were instructed that they would evaluate a photo followed by a video, without any hint of a direct connection between the two tasks. Additionally, participants were informed that valence refers to the emotional impression the photo or face evoked. After these instructions, participants were able to begin with the experiment.

During the experiment, 486 sequences were presented in random order, with 162 sequences per context condition (positive / neutral / negative): 18 facial expressions (9 female, 9 male) were presented 9 times per context condition with different context images to control for potential confounding factors such as attractiveness and gender, adding up to 27 trials per facial expression. For each emotion category, 54 images were pseudorandomly drawn from the OASIS database for pairing. Trial order was fully randomized for each participant.

**Figure 1.**
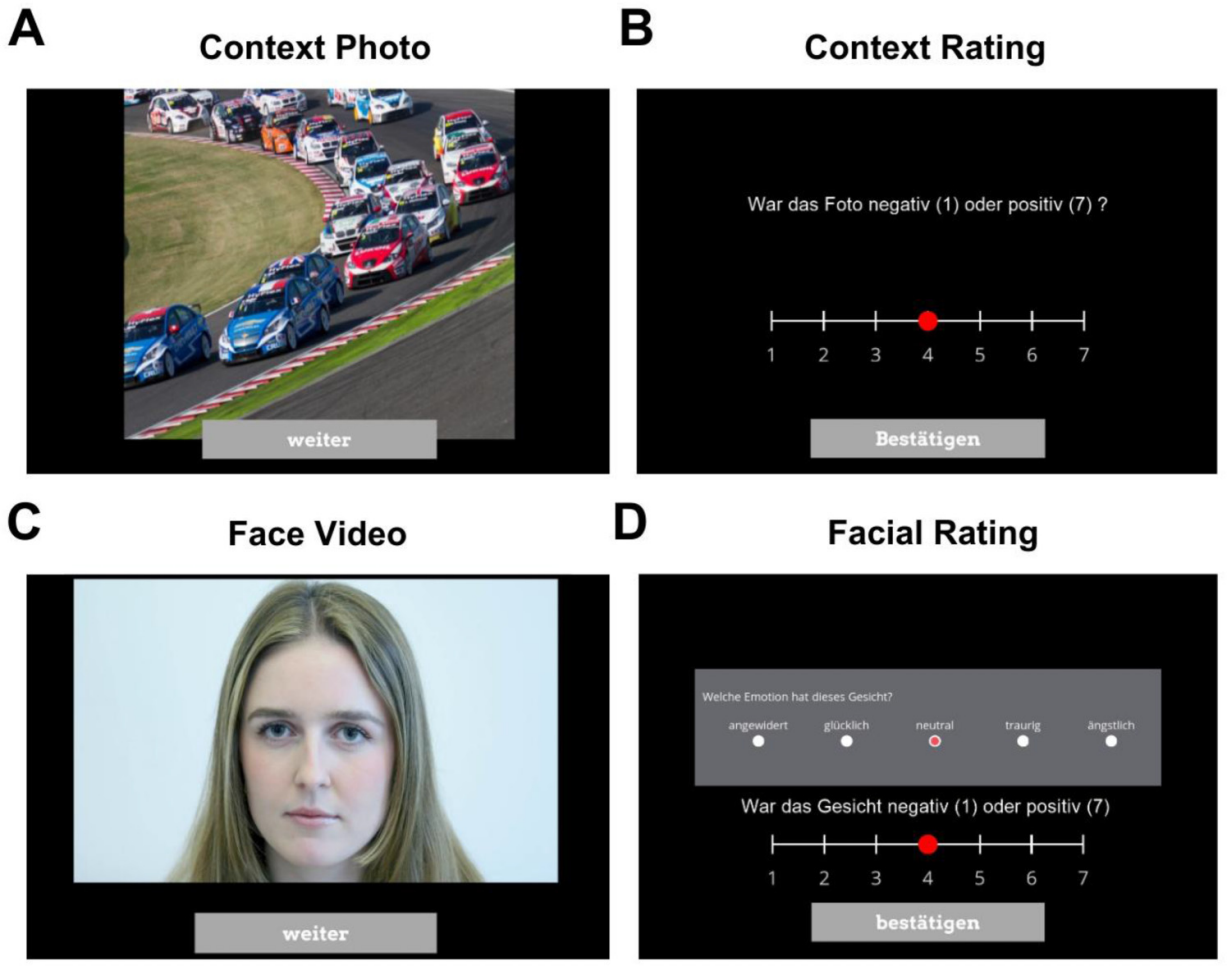
*Trial Sequence*. *Note*. Within each trial, participants initially saw a positive, neutral, or negative context photo for at least 2 s (A) before rating on a 7-point Likert scale whether the photo made a negative (1) or positive (7) impression (B). Consequently, a 2-s video of a neutral facial expression was played in a loop (C). Participants then had to assign an emotion to this facial expression (choosing between “happy,” “sad,” “neutral,” “fearful,” and “disgusted”) and also rate the face on a scale of 1–7 (from negative to positive).

An example trial sequence is depicted in Figure 1. Each sequence consisted of a context image, an evaluation of the image's valence (Was the photo negative [1] or positive [7]?) on a 7-point Likert scale, a 2-s video of a neutral facial expression (played in a loop), and an evaluation of the facial expression in terms of valence (in the following: valence evaluation)—again from negative (1) to positive (7)—and emotion (in the following: explicit categorization), where participants had to categorize the face as “happy,” “sad,” “neutral,” “fearful,” or “disgusted.” For the latter, participants were asked the following question: “Which emotion does this face display?” Participants then could select between the abovementioned categories. Participants were allowed to decide how long they wanted to view the context image and the video, provided they looked at it for a minimum of 2 s before proceeding to the evaluation. While we opted for dynamic facial stimuli to make the perception of facial expressions appear more lifelike, we relied on static images for context presentation, primarily due to the availability of validated, normed datasets such as OASIS. By doing so, we were able to retain normative consistency to be able to compare across emotional conditions in a statistically robust way.

We chose to have participants rate the context images in terms of emotional valence rather than likeability, diverging from Mullennix et al. (2019). This ensured conceptual alignment with the subsequent face-rating task, which also included a valence judgment. Likeability, while related to affective evaluation, can reflect additional sources of variance (e.g., familiarity, personal preference) that are not strictly emotional in nature but may confound the interpretation of context effects. In addition, while Mullennix et al. (2019) also asked participants to rate facial expressions along arousal, we focused on valence and emotion category only, as arousal, although a part of core affect as well, is typically less intuitively verbalizable and often more dependent on contextual and individual factors (Barrett, 2006; Russell, 2003).

### Data Analysis

For data cleaning and analysis, RStudio (Version 04.01.748, Posit Team, 2024) with R (Version 4.3.3, R Core Team, 2024) was used. Where indicated, we applied Bonferroni–Holm correction to control for the family-wise error rate in multiple comparisons and computed Cohen's *d* as a measure of effect size. Two faces were excluded from the data analysis because a non-neutral emotion was selected more frequently than neutral for these faces.

To examine the effects of context on facial valence (H1) and to check whether there were differences in the ratings of the context stimuli themselves in line with their category, we fitted linear mixed-effects models using the R package lmerTest (Version 3.1.3, Kuznetsova et al., 2017) for the dependent variables *Facial Valence* and *Context Valence* with the fixed effect *Context* (positive / neutral / negative), the covariates listed above, and a random intercept per participant. Post hoc pairwise comparisons were performed using estimated marginal means from the emmeans package (Version 1.10.2, Lenth, 2024).

To examine the effects of context on the categorical outcome emotion (H2), a multinomial logistic regression was performed using the nnet package (Venables & Ripley, 2002). The dependent variable of the regression model consisted of five nominal categories (happy, sad, neutral, fearful, and disgusted), and the reference category was set to neutral for interpretability. We performed likelihood ratio tests between the full model including the independent variable *Context* (positive / neutral / negative) and the control variables mentioned above, and the reduced model including only the control variables to assess the overall significance of our categorical predictor of interest.

## Results

### Ratings of Context Stimuli

Comparisons of linear mixed-effects models fitted indicated that context category was a significant predictor for the context valence values, $X^{2}$ (2) = 9009.80, *p* < .001, with the model explaining significantly more variance compared to the model only including the control variables and the random intercept, *R*^2^_marg_ = 47.1%, Δ*R*^2^_marg_ = 45.5%. Table 1 (left side) shows estimated marginal means of context stimuli ratings by category. Estimated marginal means of the differences between context valence ratings showed that negative context stimuli were rated significantly lower than neutral, $\hat{M}$ = −1.55, *SD* = 0.02, *z* = −62.71, *p* < .001, *d* = −1.31), and positive context stimuli were rated significantly higher than neutral, $\hat{M}=$ 1.23, *SD* = 0.02, z = 49.79, *p* ≤ .001, *d* = 1.04. These results imply that the context stimuli were selected correctly for the task at hand.

**Table 1.** Estimated marginal means of context and facial valence ratings per context.

	**Context** valence			**Facial** valence		
**Context**	***N***	** $\hat{M}$ ** **[95% CI]**	***SD***	***N***	** $\hat{M}$ ** **[95% CI]**	***SD***
**Positive**	32	5.74 [5.51, 5.97]	0.12	32	3.98 [3.67, 4.29]	0.15
**Neutral**	32	4.51 [4.28, 4.74]	0.12	32	3.93 [3.62, 4.25]	0.15
**Negative**	32	2.96 [2.73, 3.18]	0.12	32	3.87 [3.56, 4.18]	0.15

*Note*. $\hat{M}$ estimated marginal mean; CI = confidence interval; *SD* = standard deviation.

### The Influence of Context on Facial Valence Evaluation

Model comparisons also showed a small, albeit highly significant effect of context on the evaluation of facial valence, $X^{2}$ (2) = 37.50, *p* < .001, with the model explaining significantly more variance compared to the model only including the control variables, *R*^2^_marg_ = 3.6%, Δ*R*^2^_marg_ = 0.2%. Estimated marginal means of the differences between facial valence ratings showed that participants rated the valence of faces significantly more negatively when the context stimuli were negative compared to when they were neutral, $\hat{M}$ = −0.06, *SD* = 0.02, *z* = −3.60, *p* < .001, *d* = −0.08 ). Participants also rated the valence of faces significantly more positive when a preceding positive context image was shown (compared to neutral), $\hat{M}$ = 0.04, *SD* = 0.02, *z* = 2.49, *p* = .013, *d* = 0.05). Table 1 (right side) shows estimated marginal means of participants’ ratings of facial valence by context.

### The Influence of Context on Explicit Facial Categorization

The comparison of multinomial regression models with and without the variable *Context* indicated no influence of context on the perception of emotion in neutral faces: Participants did not ascribe non-neutral emotions significantly more often than neutral emotions, $X^{2}$ (8) = 9.62, *p* = .293. Consequently, the full model did not explain more variance than the model only including the control variables and the random intercept, *R*^2^_adj_ = 5.2%, Δ*R*^2^_adj_ = 0.1%. Planned comparisons (see Table 2) between the log-odds of trials with positive, neutral, and negative context stimuli indicated no significant differences for any of the emotions (all *p*s > .130), indicating that the probability that a certain emotion was selected was not dependent on context.

**Table 2.** Probability contrasts of attributing an emotion to a face depending on context.

**Emotion**	**Contrast**	** $\hat{M}$ **	***SE***	***t(36)***	***p(Holm)***	***d***
**Happy**	neutral–negative	0.003	0.004	0.86	.450	0.86
	positive–negative	0.008	0.004	2.09	.130	2.09
	positive–neutral	0.005	0.004	1.23	.450	1.23
**Sad**	neutral–negative	−0.008	0.005	−1.60	.236	−1.60
	positive–negative	−0.009	0.005	−1.83	.225	−1.83
	positive–neutral	−0.001	0.005	−0.23	.816	−0.23
**Neutral**	neutral–negative	0.007	0.008	0.9	<.999	0.90
	positive–negative	0.006	0.008	0.83	<.999	0.83
	positive–neutral	−0.000	0.007	−0.07	<.999	−0.07
**Fearful**	neutral–negative	0.000	0.003	0.14	<.999	0.14
	positive–negative	−0.002	0.0030	−0.57	<.999	−0.57
	positive–neutral	−0.002	0.003	−0.72	<.999	−0.72
**Disgusted**	neutral–negative	−0.002	0.004	−0.64	<.999	−0.64
	positive–negative	−0.003	0.004	−0.95	<.999	−0.95
	positive–neutral	−0.001	0.004	−0.31	<.999	−0.32

*Note.* Pairwise comparisons for emotion responses across different conditions. The estimate $\left(\hat{M}\right)$ represents the log-odds difference for each contrast. SE = standard error.

## Discussion

Our experiment aimed to investigate the influence of visual context on face perception according to the Kuleshov effect that proposes that a neutral facial expression will be interpreted differently depending on the context of visual scenes presented directly before or together with the expression. Earlier research has reported mixed evidence for the existence of the Kuleshov effect, with methodological variability across studies complicating interpretation. Consequently, this study adopted a design that ensures attention to context by employing explicit context evaluation and provides a clear procedure for easy replication (see also Mullennix et al., 2019, for a similar replication with static stimuli), while focusing on differences in the perception of valence (H1) and emotion categorization (H2) in an essentially neutral face. The results of our study indicate that visual context does indeed influence the perceived valence of faces; however, we found no significant evidence to suggest that visual context elicits the perception of specific emotions in neutral faces under the absence of cinematic continuity. Thus, the findings from previous studies could be replicated for facial valence evaluation, but not for explicit categorization of emotions, potentially suggesting that cinematic continuity is needed for the Kuleshov effect to arise.

### Visual Context Influences Facial Valence Evaluation

Importantly, our comparisons showed that both positive and negative contextual information did in fact exert a respective influence on the evaluation of facial valence. In theory, the differences in ratings we observed could have been caused by differences in context valence (e.g., neutrally labeled pictures being perceived as more positive than positively labeled pictures). However, we were able to eliminate this potential confound through the appropriateness in ratings of the context stimuli, as contexts of the respective category were also rated accordingly. Our results are also in line with previous studies that state that context can systematically sway how observers interpret otherwise ambiguous or neutral facial expressions. Wallbott (1988), for instance, reported that film and television clips influenced observers’ emotion attributions in a manner consistent with the surrounding context, suggesting that editing choices could indeed modulate the perceived affect of a neutral face. Similarly, Mullennix and colleagues (2019) found that standardized still images, varying in positive or negative valence, affected participants’ ratings of facial neutrality, thereby showing that contextual cues are able to shift emotional interpretations of facial images. Barratt et al. (2016), using standardized stimuli and measuring valence on a 9-point Likert scale, also reported significant contextual effects on facial valence evaluation. Similar results were obtained by Calbi et al. (2017) using a 5-point Likert scale for each face that measured pleasantness (a direct operationalization of valence); these findings were replicated in a 2019 follow-up study (Calbi et al., 2019). Cao et al. (2024), using authentic film footage, found convergent behavioral and functional magnetic resonance imaging evidence that demonstrated the robustness of these effects.

Our present findings also reinforce a longstanding proposal from dimensional models of affect (Russell, 2003) that valence can operate as a “gatekeeper,” whereas explicit categorization requires stronger or more unambiguous cues. Dimensional models argue that the fundamental information extracted from a face is its affective dimensions, that is, valence and arousal, forming the basis of emotional experience. Under this view, humans initially register how pleasant or unpleasant a face is and only later construct a specific emotion label; behavioral and neurophysiological evidence indeed supports the idea of rapid and automatic valence processing (de Gelder et al., 2006; Eger et al., 2003).

### Context Does Not Change the Perception of Emotions

As results of our multinomial regression show, context did not exert a significant effect on the perception of emotions in neutral faces. Although participants often selected an emotion other than “neutral,” there was no significant difference between the two generalized linear mixed-effects models with and without context as an independent variable. Our results therefore differ from some of the previous findings, where this effect was found (e.g., Barratt et al., 2016; Calbi et al., 2017; Wallbott, 1988).

A key difference between our design and the designs just mentioned is that their designs preserved cinematic continuity contrary to our dyadic front-facing design that has intentionally removed elements pertinent to past studies (see Introduction above). The present null finding for explicit emotional categorization therefore supports the idea that continuity editing and shared spatial context are critical for moving observers beyond a simple valence judgment and toward assigning a specific emotion label. For instance, Mullennix et al. (2019), in a design similar to ours but using static faces, still found (weak) categorical effects, emerging, however, primarily for disgust that is considered a particularly strong emotion. Interestingly, even with short face videos, categorical effects could not be induced once continuity editing was not a part of the experimental design. Studies in which continuity editing was present actually significantly shifted the distribution of chosen emotion categories; this was the case for Barratt et al. (2016), where participants could select a single basic emotion out of seven or a neutral category, as well as for Calbi et al. (2017) where participants were forced to choose between six basic emotions plus a neutral category.

An intriguing partial exception is the study by Calbi et al. (2019): Here, the authors found that even though there was a significant behavioral effect, EEG results still indicated that the face was evaluated as neutral. Conversely, Prince and Hensley (1992) found no context effect on explicit categorization at all. While they concluded from their findings that the power of deliberately altering emotions through context was less robust (if at all present) than suggested by the “legend” of the Kuleshov effect, their conclusions must be viewed in light of three limitations: the study used a single-trial montage, the participants were already familiar with the Kuleshov effect, and only categorical (not dimensional) ratings were collected. These properties of the design likely speak for low statistical power and limited generalizability.

Our study adds to this controversial pattern by showing that while contextual framing affects perceived valence, it does not alter categorical emotion attribution. While this finding may be in line with previously mentioned accounts of dimensional affect models that see the attribution of emotion as a slower, more conceptually driven and deliberate process compared with the attribution of valence (Gao et al., 2022), the present dissociation might, however, rather reflect the lack of narrative continuity and cinematic realism in our paradigm, as both behavioral and neural evidence presented point to the presence of an effect of explicit context on categorization. The usage of static context images, which, contrary to the dynamic context stimuli used by Barratt et al. (2016), could also lead to limited emotional resonance. The omission of such elements so typical to filmmaking may therefore have reduced the likelihood of viewers constructing a narrative in which the face is interpreted to be reacting to an emotionally meaningful event.

### Strengths, Limitations, and Future Directions

The present study has had multiple strengths that we were able to capitalize upon: These include the usage of a standardized database for context images, the use of videos (and their prescreening by multiple subjects), and a theoretically and methodically robust experiment minimizing confounds and statistical power issues. There were, however, also a number of limitations.

First and foremost, while our design increases internal validity by the omission of typical elements of studies looking at the Kuleshov effect such as gaze-contingent editing and continuity between context and face, it also constrains ecological validity. By omitting these conventions that are central to how viewers naturally interpret film sequences, the present paradigm may not capture the full mechanism of the Kuleshov effect as it is classically understood. Future studies may benefit from systematically reintroducing these elements to examine whether doing so reestablishes conditions under which the Kuleshov effect, as classically defined, more robustly emerges.

In combination with neutral context, participants perceived emotions in faces that were supposed to be neutral in 29.03% of trials, indicating that neutrality might not have been fully achieved to begin with. One possible explanation for the lack of a significant effect on emotion is that any effect of visual context on emotional perception was obscured because the supposedly neutral faces may have been not truly neutral. However, it is important to mention that face videos were prescreened by a committee of seven participants to distinguish neutral from non-neutral facial expressions. The small size of this committee, however, lowers confidence in stimulus neutrality (as it may have contributed to the exclusion of two faces at a later stage of the analysis). Future research with greater resources should employ a larger number of neutral faces that are thoroughly and reliably tested for emotional neutrality. Employing automated facial expression analysis tools during prescreening may help minimize any residual emotional cues in the videos.

The length and repetitive nature of the task may also raise concerns about fatigue effects. While we did not observe behavioral indications of disengagement, it remains possible that performance was subtly affected by declining attention over time. Of note, however, in typical cognitive psychology experiments, the effects of fatigue are less prevalent than learning effects. Nevertheless, future studies could include attentional checks or incorporate motivational elements to more directly assess potential fatigue-related variance.

Limiting ourselves to the assessment of behavioral variables can be seen as both a strength and a limitation of the present design; while a behavioral-only approach is quite efficient and generates important insights in a fraction of the time compared to, for instance, a neuroimaging experiment, it also leaves a large number of corresponding variables untouched. Incorporating physiological or neuroimaging measures together with self-report data might therefore provide deeper insights into the underlying mechanisms of the Kuleshov effect.

## Conclusion

In summary, our study confirms our first hypothesis that emotionally valenced context shifts the perceived valence of an otherwise neutral facial expression. We found that the more positive the context was, the higher the valence rating for neutral faces, and vice versa; however, we found no conclusive evidence for the notion that visual context could induce changes in the explicit categorization of emotion in faces deemed neutral. While the former finding is in line with previous studies, the latter is not. This is possibly due to the specific features of our dyadic design inserting an explicit context-rating phase and thus removing the spatial continuity and return-gaze cues typical of classic Kuleshov sequences; these changes may therefore likely weaken (or in our case even abolish) top-down mechanisms required for explicit emotional categorization.
